# The Transactions of the Provincial Medical and Surgical Association

**Published:** 1835-07-01

**Authors:** 


*The Transactions of the Provincial Medical and Surgical Association.* Vol. III.—London, 1835. 8vo. pp. 472, xxvi. and xii.

The first paper in this volume is a retrospective address, delivered by Dr. Conolly, at the second anniversary meeting of the Association, and is characterized by the accurate judgment and flowing style which distinguish his writings: yet, when he remarks that, in the study of diseases of the brain, of the chest, and of the abdomen, all must acknowledge how much we owe to foreign cultivators of medicine, we would ask, are not the physicians of the Parisian hospitals (whom he especially cites,) far more behind us in practical knowledge than we are behind them in morbid anatomy? Do we not find, in the French journals, that Andral is beginning to learn the advantages of purgatives in diseases of the chest, while others are becoming acquainted with calomel and the benefits of mercurial inunction in ophthalmia? Are not the bicarbonates of soda and potash put down as new remedies in Magendie's "Formulaire"? Dr. Conolly proceeds as follows.

"There is still another department of medical study and practice in which the French have done much, and we have done little. In the clinical study of mental disorders no advance can be reported: it is yet entirely overlooked in English medical education. Lectures are delivered, and treatises are written, but practical observation is limited to the officers of institutions for the insane. Hospitals for lunatics are closed to the student, and the knowledge of mental affections makes little or no progress. As a consequence of this, not a year passes without some notorious case of insanity being brought before the public, in a manner reflecting little credit on the conflicting medical authorities; whilst instances of the injudicious or improper confinement of patients are not unfrequent, and such as neither the visitors of asylums, nor the metropolitan commissioners of lunacy, nor the Lord Chancellor, are found to have, or to exercise, any power over. To the same negligence of this whole subject, it must be attributed that we have so few aids to a knowledge of the conditions of the brain in the various forms of insanity, that the pathology of insanity is so uncertain and incomplete, and the treatment so generally empirical. Our larger lunatic establishments have contributed little or nothing to our knowledge; and, in many of the private lunatic houses, there is too much reason to fear that therapeutic measures still obtain very little attention. When the public establishments are made (as, under proper regulation, they might be,) schools of instruction, we shall learn how much our practice in these distressing disorders may be improved. The phrenologists have attempted, without much encouragement, to throw some light on the causes of insanity; but, although the truth or error of their opinions on this subject is closely connected with the most interesting speculations that can employ the reflective powers of man, their views have, in very few instances, been examined in a philosophical spirit, or indeed with candour, either by physiologists or pathologists. There is reason to hope that, as a result of the humane and enlightened management of the large lunatic asylum at Hanwell, under the superintendence of Dr. Ellis, this branch of practice may hereafter be more satisfactorily spoken of." (P. 17.)

After mentioning the Bridgewater Treatises, and the advantages which the general as well as the scientific reader may derive from them, our author passes on to the late improvements in the remedial means employed in medicine and surgery. Among these, the use of iodine in scrofula, of strychnia in paralysis, and lithotrity, are the most important.

Dr. Conolly gives a very flattering account of the periodicals dedicated to medicine.

" In the medical periodical literature of this country, which exercises considerable influence on the whole profession, few recent changes have taken place. We may observe that, for the most part, such publications are devoted to the useful but unambitious task of making abstracts or selections from new works, for the benefit of hasty readers, or of those not likely to see the originals : an office which, upon the whole, they perform with ability and fairness. Whatever objections may exist to such a plan, it evidently leads to the ready circulation of much professional knowledge throughout the world. Some of our journals are more strictly chronicles of passing events; and such seem particularly required at a time when questions of medical polity are daily discussed. If not altogether free from personalities and illiberality, this portion of the medical press is yet, for the most part, conducted with much intelligence, and by men of integrity and honour. The Quarterly Journal of Medicine is to be noticed as a new publication, and it sets out with considerable claims in the department of critical writing, in which English medical journals have been generally defective. The long established Medical and Surgical Journal of Edinburgh, associated in the minds of so many readers with their earliest studies, still maintains, especially in its critical notices, the high rank which it has so long possessed." (P. 22.)

Among the various topics discussed in this Address, some of which we must necessarily pass over, one of the most interesting is that of Self-supporting Dispensaries. Dr. Conolly remarks that, " unfortunately, also, in some towns, institutions have been conducted professedly on Mr. Smith's plan, but so curiously perverted from it as entirely to exclude that self-dependent principle which is the very soul of the new system." (P. 35.) We fear that this is the case in London, and that there is not yet one dispensary which is really supported by he contributions of those who are to be benefited by it.

In his observations on the transactions at the Aldersgate-street Dispensary, our author coincides with all thinking and honourable men; but his sentiments are so vigorously expressed, that we should wrong our readers were we to omit them.

"Among the medical events of the past year, it would be wrong to pass over an indication of independent feeling, redeeming amidst too many examples of voluntary professional degradation. It had arisen from the eagerness of medical men to obtain appointments to public institutions, that, on the announcement of any vacancy, the promoters of charities for the benefit of the sick poor were exposed to all the excitement and all the manœuvring attendant on a popular election. There were not, it is true, the treatings, the drinking, and the ribboned processions, but only the duller and deeper spots of such humiliating scenes: the abject solicitation, the secret circumvention, and the manufacture of votes. By the last-mentioned device, claims established by honourable services were put aside, the real interests of charities trampled upon, the steady supporters of a charity outvoted, and the nominal supporters of one year indulged in a most pernicious patronage. It is gratifying to have to record, although I shall do it without comment, an example of manly opposition to this system, by the medical officers of the Aldersgate-street Dispensary,* which called forth prompt expressions of professional approbation, and went far to extinguish the corruption of medical elections for ever." (P. 35.)

A part of this discourse is naturally dedicated to the memory of professional men, recently deceased: the obituary of our author contains Dr. Darwall, M. Boyer, Sir W. Franklin, Dr. J. Gordon Smith, Sir Gilbert Blane, Dr. Henry Gaulter, and Dr. Becker. It would be difficult to point out a writer better qualified for this task than Dr. Conolly: his short accounts are terse and satisfactory, while the biography of Dr. J. G. Smith, which it would be unpardonable not to quote, shows great discrimination of the nicer shades of character, and the undefined debatable ground which separates human frailty from human virtue.

"When looking over the leaves of one of the periodical works some months ago, I saw an announcement of the death of Dr. John Gordon Smith, and the distressing fact was stated that he died in prison. No tribute to his memory seems to have been paid by the publications of a profession of which he was once a useful, and even a distinguished member. No friendly hand has recorded the merits of one of the most unfortunate of men. A sense of justice,

* "Drs. Clutterbuck, Birkbeck, Lambe, Roberts, Mr. Salmon, and Mr. Coulson."

as well as a feeling of generous compassion, demands something more. When this ill-fated physician published the first edition of his Treatise on Forensic Medicine, his mind was vigorous and healthy, and that work was accepted and long quoted as one of high authority. In every successive edition and in most of his subsequent publications, may, I think, be traced, among much that is acute and striking, the marks of a mind somewhat declining towards disease. And such was really the case. He had become subject to attacks of violent and obstinate irritability of the stomach, and to these attacks invariably succeeded periods of great mental excitement, the subsidence of which was soon followed by a return of the gastric irritability. By these alternations, which afforded him little repose, his mental and bodily health became, in time, visibly impaired. His self-control was much diminished, and he fell into eccentricities in writing, in conversation, in manners, and in dress. He made repeated attempts to deliver lectures on Medical Jurisprudence, but the commencement of a course always seemed to aggravate or induce his mental excitement, and I doubt whether he ever completed one. He began to perceive that he was neglected, and he sometimes imagined that he was insulted, when no insult was intended. He became suspicious of his friends, and bitter, as far as words went, against his enemies. The limited state of his circumstances made his professorship in the London University an important consideration to him, yet he resigned it in haste, on a supposed affront; and the immediate filling up of the appointment too clearly shewed him that his retirement was not regretted. I have letters from him, written at that period, in which he calmly and affectingly states his melancholy situation; laments his irritability and its then irretrievable consequences; and expresses his painful disappointment at being, as it were, excluded from the arena of medical teaching, just when the great desire of his life was accomplished, by the admission of his favourite science as an acknowledged part of medical study. He had laboured many years without reward; and when the reward seemed within his reach, he was consigned, he felt, to hopeless indolence. I saw Dr. Gordon Smith at that time frequently, and I beheld with concern a man of a mind of great acuteness, of considerable literary acquirements, of much wit, and great powers of conversation, and of a disposition singularly warm and confiding, but passing into obscurity, and with the prospect of ruin before him. Embarrassed circumstances, sickness, insanity—the mad-house and the prison—filled up, I greatly fear, the interval which followed between that time and the merciful close of his mortal existence. Yet to him the science of Medical Jurisprudence will always remain indebted; and it ought never to be forgotten that he was one of the first to shew, and zealously to advocate, what all now acknowledge, its usefulness and dignity. It is said that on more than one occasion he was instrumental in saving the life of an accused person, really innocent: if such were the case, that record on his tomb may well outweigh a life of pain and disappointment; and doubtless it will there be remembered where good men's deeds are not forgotten, and sorrow can exist no more." (P. 38.)

We next come to *A Report on the Chemistry of the Blood, as illustrative of Pathology,* by Mr. Egerton A. Jennings. The following are some of the more prominent points in this interesting paper.

In *jaundice,* the blood contains both the colouring matter and the resin of the bile.

"The analysis of the blood in *rheumatism* has not, at present, afforded us any satisfactory information respecting that disease. The intensely arterial colour of the blood in rheumatism, the pulsatory motion with which it is frequently expelled from the vein in bleeding, and the uncertainty of the effect of bleeding on the disease, all point out a marked difference between the character of blood in rheumatism and in fever or inflammation; yet, when we notice the extreme mobility of rheumatic pains, which appear to run wherever the blood is distributed; when we see the application of a few leeches to the part entirely remove the pain, which shortly reappears in some other place; and when we see a single free bleeding sometimes stop the disease, as if, by diminishing the fluid, it proportionably cut off the stimulus which occasioned all these migratory irritations; we cannot (as M. Andral has remarked,) but look to the blood as a material agent in the production of rheumatism." (P. 72.)

But the most marked alteration of the blood in rheumatism is in the fibrine: in this disease, however far bleeding may be pushed, the buffy coat remains.

In *gout,* the blood is loaded with earthy phosphates and highly azotised products.

In *dropsy,* the blood is often watery; but the disease may be occasioned by the greatly increased quantity of the blood, its quality being in no way impaired. (We suspect this to be extremely rare.)

Drs. Bright, Bostock, and B. Babington, have found that, when dropsy is attended by albuminous urine, the blood is deprived of a large portion of its albumen, and contains urea, the quantity of the latter being diminished in the urine. Other chemists assert that the diminution of albumen in the blood, under these circumstances, is by no means constant.

In *chlorosis,* the blood is very watery, the proportion of crassamentum to serum being less than one third of the healthy average. The deficiency in colouring matter is singularly great: a thousand parts of blood, which ought to contain 133 parts of colouring matter, in one chlorotic case contained only 52, and in another only 48·7.

Now, "Mr. Brande* states that menstrual blood consists of a concentrated solution of colouring matter in diluted serum; and here we find a diminished proportion of colouring matter in the blood, attended with suspended menstrual secretion. The colouring matter of the blood is the portion from whence we obtain the iron. If the colouring matter be diminished, the proportion of iron must be diminished also. The administration of iron proves the most efficient mode of restoring menstruation. Can it, then, be doubted that in chlorosis menstruation is suppressed, from the absence of sufficient colouring matter in the blood to supply the secretion; while the administration of iron restores menstruation, by first increasing the proportion of colouring matter in the blood." (P. 77.)

*Scurvy* is another disease in which the chemical character of the blood is much altered: it is probably produced by an excess of saline matter in the blood, depriving it of its power of coagulation; and hence acids cure the disease, by restoring its power of coagulating. (This does not account, however, for the remarkable superiority of the citric to other acids in the treatment of scurvy.)

In *cholera*, there is a deficiency of water, as well as of alkaline salts in the blood.

As bleeding is seldom required in *scrofula*, there is no recorded analysis of blood in this disease; but, in the few instances where Mr. Jennings has examined the blood, the proportion of serum to the crassamentum was considerably increased. Pregnancy checks scrofula, because in pregnancy the blood undergoes important changes, and contains an increased proportion of crassamentum, and particularly of fibrine.

In *carcinoma*, the morbid matter is probably formed in the blood, and "then deposited in the molecular structure of organs, in the same manner as the nutritive element of the blood, or is poured out on free surfaces on serous membranes, in a manner similar to the natural secretions." (P. 83.)

We must pass over the rest of this very instructive paper, and content ourselves with some of the author's conclusions.

"From the facts collected in this Report, we may conclude, 1st, That, in many diseases, the blood has been proved to undergo material alterations in its composition; and, in numerous other affections, there is good reason to suppose such changes to take place. 2d. That many of our medicines enter into the blood, and probably produce their remedial effects through that medium.

"Our knowledge of what medicines act by producing changes in the blood, and what by other means, is at present very imperfect. Some have been pointed out which undoubtedly enter that

* Philosophical Transactions, 1812, p. 113.

fluid, and probably through it produce their effects. Others again, as the salts composed with vegetable acids, and the large class of medicines taken from the vegetable kingdom, are some decomposed, and others digested, in the stomach. A third class, the sulphates and phosphates, the stomach possesses no power over: they are not decomposed by it, and mostly pass off by the bowels, on which they produce a purgative effect." (P. 91.)

Mr. T. TURNER has contributed a long paper on the present state of *Anatomy*, which might form a useful manual on the subject, if carefully rewritten. The following is Mr. Turner's account of the otic ganglion.

"That the ganglia (not merely those of the great sympathetic system,) in the vertebrata are subservient to involuntary actions, or functions, is, I think, a plausible opinion, and appears to be strikingly corroborated, or at least strengthened, by one of the presumed functions of the otic ganglion, recently discovered by Arnold. This body, which is situated near the cartilaginous part of the Eustachian tube and the origin of the tensor tympani, forms communications with the fifth and the ninth, by means of the tympanicus, and, lastly, by means of the latter with the facial and auditory nerves; which anastomoses are of the utmost importance in a physiological point of view. Many nerves derive their origin from this ganglion, but the most important of them is one which ramifies in the tensor tympani muscle. In anatomical character, the ganglion oticum is analogous to the ophthalmic ganglion; and, in office, it bears the same relation to the membrani tympani that this last ganglion does to the iris. To comprehend this, it is necessary to understand that it is believed that the membrane of the drum has a double function,—namely, the propagation of sound, and that of being the means of protection and a *moderator* to the nervous ramifications in the interior of the ear. In the former office, the membrane vibrates from the fluctuation of the air; but, in acting as a means of protection, the membrani tympani does not merely keep off the immediate effect of air and other injurious influences, but moderates the too-powerful action of sound on the internal ear. The discovery of the ganglion oticum is an interesting point in modern anatomy; and Mr. Tyler, who has translated Arnold's paper from the German, is entitled to the best thanks of the profession." (P. 154.)

Dr. SYMONDS gives an account of the *Cholera* as it appeared at Bristol in 1832, in which the reasons for and against its contagiousness are well summed up.

First, *contra:*

"(*a*) There was not the slightest reason to suspect that the person first attacked on the 11th of July, had been exposed to infection.

"(*b*) The disease broke out in distant parts of the town, without any communication being traceable, and in some instances almost simultaneously; in scarcely any instances did it appear to spread by continuity.

"(*c*) Even in a limited space, as in one street for instance, it would rage, at the same time, in sets of houses, comparatively remote from each other; the intermediate dwellings being quite free, although the circumstances of intercourse between the inhabitants were equal.

"(*d*) It often happened that individuals, having staid long enough in a diseased locality to contract the malady, did not introduce it into their own neighbourhood, in which they lay ill and died; and the same was true of many who fled from an infected district, and were seized, in their new abode, two or three days afterwards. The case of the sailor who had been in London, and died at the Hotwells in the month of March, is a remarkable instance of the kind.

"(*e*) Not a single medical man was attacked with cholera, although most of the profession, engaged with the disease, experienced a diarrhœa, which they attributed to the combined effects of fatigue and atmospheric influence." (P. 182.)

Secondly, *pro:*

"(*a*) Scarcely a single nurse, or other attendant, at the hospital, escaped the disease in some form or other: the men employed in carrying the sick in baskets to the hospital, were extremely subject to the disease, and particularly the bearers of corpses; individuals who, in the agony and distraction of grief, had clasped the dead bodies of their friends or relatives, often sickened very shortly afterwards.

"(*b*) Persons approaching the bedsides of very malignant cases, or passing the corpses carried out of the wards of the hospital, were sensible, not only of a very peculiar odour, but also of sudden nausea, *malaise*, an indescribable gloom, and frequently spasmodic twitchings of the muscles of the face and extremities. Patients, to all appearance, convalescent, would suddenly relapse, soon after the introduction of a very bad case in the next bed, as if from a reception of a second dose of the poison.

"(*c*) The persons first attacked in a part of a parish, had, in some cases, been in communication with the sick elsewhere.

"(*d*) A girl became ill, and was removed to Thornbury, about nine miles distant; her complaint proved to be cholera, but she recovered. Her mother, however, who had not been in Bristol, fell ill of the same disorder, and died. The malady, did not spread in consequence, as the residents adopted very strict measures of seclusion." (P. 183.)

Mr. Rumsey's account of an *Epidemic Scarlatina,* which appeared at Beaconsfield, in 1832, makes us almost indulge in an observation like that of the connoisseur in the Vicar of

Wakefield, who used to remark, that the picture would have been better, if the painter had taken more pains. This essay should evidently have been rewritten: it is in an undigested state; but we will not insist upon this point, as we have *quod accusatori maximè optandum est, confitentem reum.* Mr. Rumsey regrets the want of time, &c. The account, however, is practical and praiseworthy, as will appear from the following extracts.

"The symptoms ushering in the disease, were a slight sense of soreness in the throat, the tonsils and velum pendulum being red, often with a yellowish slough, and sometimes with a coat of aphthous-like exudation on the surface; the exudation going off, would leave sloughs more or less deep. There was more or less bleeding from the nose, giddiness, pains of the legs, head and back, with frequent retching. Sometimes the mere food would be thrown up, and at others, the food tinged with green bile. In one case, which proved rapidly fatal, with symptoms of great prostration, it was ushered in by aphthæ in the throat, without any symptom to distinguish the disease for six and thirty hours afterwards; so, that it had proceeded to almost a fatal termination, without the character of scarlatina.

"The pulse would usually be about 120 after the first day. The tongue being at first of a greyish white colour, and moist; often on the second day, and later, having a browner broad stripe down the middle to the tip, with dryness. The eruption would appear in about forty-eight hours, often sooner, after the disease commenced, being preceded by an unequal redness of the face and arms, without the stigmata of a rash, or the equal colour of a flushed skin: so that by this patching redness, it might sometimes be predicted that an individual would soon be the subject of the disease, before he had actually become so.

"In several cases the bursting out of rash was accompanied by well-marked nettlerash blended with it. The glands under the jaw were tender from the earliest stage, a circumstance calculated to assist the diagnosis, in such cases as might be confounded with measles. The conjunctiva was red, and in many cases a defluxion of thin ichor was poured out from the nostrils. It would often happen that very early, as on the first, second, or third day, there would be a great and alarming sense of weakness, seeming to be a degree of collapse, rather than debility produced by exhausting symptoms, or the known and usual depression of fever. This depression was known to go off without the assistance of stimuli, in one or more cases; which I mention as favouring the opinion that it differed from exhaustion. It is an alarming feature in the disease, and often bids defiance to our most vigorous employment of cordials. The weakness which has its corresponding, and intelligible causes, is one thing: that which sets our powers to explain it at defiance, and is related, probably, to some principle connected with the hidden physiology of nerve and which is called collapse, is another; and their distinct nature should be seen, in order to enable us to form a rational prognosis. In other cases the depression was too excessive to admit of any delay in the most vigorous use of cordials. The tongue would acquire a thick coat, which in one instance was very rapidly formed, and bore a resemblance to aphthæ. There would be, in some cases, a little hoarseness and cough, and often, on the decline of sore throat, a very troublesome and irritating cough; but a most strikingly smaller propensity to catarrhal affection than is seen in measles. The urine was sometimes copious and pale during the first twenty-four hours. The appetite upon the access of the disease ceased. On the second and third days the skin became hotter, but seldom, I apprehend, exceeding 103° of Fahrenheit. The inflamed throat, in the less favourable cases, would assume a dark red hue, its vessels readily giving way, and bleeding, if touched with a spoon. If the tonsils were enlarged and firm, of a light red colour, suppurative inflammation was to be expected, more than malignancy, and affording a more favourable prognosis. Viscid and troublesome mucus would occupy the fauces. Observation confirmed the otherwise reasonable conclusion, that a bright red eruption was more favourable than that of a livid hue. In some, the eruption would be coarse and patchy, more or less distinct, growing browner on the fourth or fifth day, and then going off. I observed in several instances, and have formerly noticed the same fact, a return of a well characterized eruption, more than a week after its disappearance." (P. 196.)

"I observed nothing in the alvine discharges peculiar to this disease, or differing from what any continued fever might present. In one case the motions had a dark-brown hue, with, as it appeared, an overload of bile; in which I gave, and repeated, an active purgative, when the subsequent motions became of a proper colour; the previous evacuations having had no striking influence upon the case. It happened in several cases, as I have before, on other occasions, observed, that violent cholicky pains would come on. These have been sometimes relieved by the warmth of clysters; I believe, also, that they are relieved by antispasmodics; yet I would earnestly recommend, upon their occurrence, a careful investigation into the accompanying symptoms, lest they should, in certain individuals, have the inflammatory character, and end in corresponding consequences. There is no doubt that this remarkable pain has a peculiar relationship to the disease. I have witnessed its occasional connexion with it ever since I was a student in medicine, and for the first time in the year 1811." (P. 200.)

"To lower the temperature of the throat externally by evaporation, appeared to me to be preferable to blisters, and highly grateful to the patient. I never met with a clear demonstration of the usefulness of blisters, but often of their trouble to the patient: nevertheless, I occasionally employ them. I include, in my remedial treatment, a constant confinement to bed, and a careful and frequent observation of the temperature of the body; taking off or adding covering; drawing and undrawing curtains; admitting or excluding the light; renewing or extinguishing the fire; keeping it clear, without smoke; enjoining, with peculiar urgency, the recumbent position, as soon as any characters of great debility have appeared. I consider it to be very important to admit so much fresh air as that the rooms shall never be close; and to this end I advise the removal of carpets and superfluous furniture. The rooms should be wiped, both walls and floor, for the purpose of removing dust, as the dust, I think, has an effect in disqualifying the air for respiration. In the advanced stages of the disease, and under the circumstances of collapse, a most rigid economy of strength should be used. The throat should be inspected without much disturbance to the patient; and children should not be struggled with when they resist, unless it be clear that there is a great necessity for inspection. Opiates, to procure rest and allay irritation, as circumstances dictate, are useful. It is not uncommon to find, in the early stage of the disease, before the strength has been exhausted, a pulse of full volume, which, with the accompanying hot skin and flushed face, might, in other circumstances, suggest the necessity of general bleeding. It would be right, however, in this disease, to subject these symptoms to strict scrutiny, in respect to other attendant circumstances and appearances. It should be ascertained whether the sensorium is affected by any the slightest deviation from its usual integrity; whether the memory fails, or a remark is made twice over; whether there is any tremor of the muscles, or subsultus tendinum; whether the erect position of the trunk, in bed, hurries or weakens the pulse, or the countenance grows pale under it; whether all or any of these circumstances happen in the slightest degree. Such a test of diminished strength is the best that I know, and should, in my opinion, if such characters exist, guide the practitioner to avoid lowering the system by active or negative means; especially if there be an epidemic, which may have exhibited cases in which great and serious depression of strength had spontaneously come on. I have witnessed the mischievous effects of lowering a patient too much in pneumonia, where the case has been accompanied by sensorial disturbance and muscular tremors, even in the athletic and youthful. There is, then, in scarlatina, a state which might be called a mimicry of plethora and strength, while the disease is, nevertheless, on the verge of collapse, and the pains to be employed for the discrimination cannot be too carefully insisted upon." P. 206.

Mr. James has contributed "*Observations on some of the Causes which influence the Formation of good or bad Stumps, in certain Cases of Amputation of the Thigh.*" He remarks, that it is still a well-grounded complaint that defective or projecting stumps are not unfrequent, and quotes Mr. Mayo, who observes, that, "after amputation of the thigh, the surgeon is often disappointed at finding that, careful as he may have been to guard against this particular evil, the bone projects." (*Med. Quart. Rev.*, vol. ii. p. 411.)

Defects in the operation form the first class of causes which may produce bad stumps, as when the section of the bone is made too near the knee: or the difficulty may arise from the unhealthy state of those tissues of the limb, which are capable of affecting the formation of a good stump. Thus, the skin may be loose and redundant, or there may be an excess of fat in the integument; or,

"Thirdly. The muscular flesh of the limb may be much changed in various ways: it may be simply emaciated; or it may be swoln and agglutinated, either from acute or chronic inflammation. In the first case, a good stump will hardly be formed, unless the bone is cleared high up; in the second, from want of contractility of the muscles, a similar proceeding will be essential: but it is to another degeneration that I now particularly solicit attention, in which, from long disuse, (the consequence of old-established disease in the knee or leg,) scarcely any trace of muscle is left in the lower part of the limb: the fibre has been for the most part absorbed, and either we find mere fat in large quantity, or intermixed with a little fibre; or, what is not uncommon, the muscle converted into a sort of firm, gristly, semi-adipose matter, perfectly incapable of contracting under the knife. Now, in such cases as these, I cannot but suspect that either of the methods practised by M. Dupuytren or Sir C. Bell, or those of many other surgeons, would fail: they are all based upon the principle that the muscles contract as they are divided, which is not at all the case here: the only mode of forming a good stump, I apprehend, in these cases, is by clearing and sawing the bone high.

"The muscles may not have degenerated, but another change may have taken place in them, depending upon a long-continued position of the limb: thus, where the knee has been for a considerable period fixed in a bent position, the flexors will have their distal points already much approximated, and will not shorten in any great degree after they are divided; while the extensor muscles, which have been in the same proportion on the stretch, will retract much more: if, therefore, both are divided in the same plane, the latter will be deficient, the former projecting. The separate division, at different heights, of these two classes of muscle, will enable us to overcome this defect; and the position of the limb, after the operation, may be so regulated as to give an advantage to the defective portion.

"Fourthly. It not unfrequently happens that the state of the bone makes an essential difference in the operation. When we are obliged to amputate for necrosis of the thigh, (affecting the knee-joint, as it often does,) it not unfrequently happens that we are obliged to divide the bone where, with the swoln periosteum, and the adjacent hard cellular tissues, it has a much larger bulk than common; the muscles around being at the same time much agglutinated. In these cases I must express my belief that it is most essentially necessary to save an ample quantity of integuments, as our only sure resource for covering the bone: we may, or may not, succeed, in the progress of the operation, in preserving muscle enough, but of integument we are secure; and, although it is not the best material, yet it is infinitely better to protect the bone with this, than to leave it in any degree bare, which will inevitably produce a multitude of evils. If we can save the integument without detaching it from the subjacent muscles, (a plan much condemned by many authors,) so much the better; but, if not, the knife must be used." (P. 223.)

In relation to the frequent fatality of the operation, Mr. James says:

"In considering the question, it is necessary to advert to the nature of this particular wound, which not only involves the ordinary soft parts, but also bone; and, if the matter be viewed in this light, we shall perhaps perceive little difference between the wound resulting from an amputation, and that of a compound fracture. It is true that, in the stump, we ought to have no laceration of the soft parts; but, on the other hand, they are often in a very unhealthy condition. It is also true that we have no spiculæ of bone to produce irritation; and, although the injury done to that part by the saw is very great, yet it is much more limited in extent than in fracture. I apprehend, however, that the formidable results often consequent on this operation, if the wound passes into any considerable degree of inflammation, may be much explained by the obvious analogy with the accident alluded to above: hence, as in compound fracture, the case often does worse if the surgeon persists in his endeavours to keep the wound closed, (the first attempt at union being defeated,) than if he throws it open: hence the severe constitutional irritation, the disposition to phlebitis, &c. Either injury is liable to causes of aggravation peculiar to itself: in the compound fracture, every motion disturbs the process of ossific union essential to the cure, and may cause the shattered bone to irritate the soft parts: in the stump, this is not the case; but then each movement may displace the loose muscles, ready to shrink up, and destroy the recent adhesions. In the compound fracture, it is the confining and collecting of matter which produces the worst symptoms: the same thing will happen in stumps, if early care is not taken to give it vent. Our true advantage in amputation is, that we can, if we choose, always effect this, while in compound fracture we often cannot.

"There is one circumstance which strongly evinces the similarity of the process, in cases of bone divided by the saw, and those where it is fractured,—namely, that, in many amputations, much osseous matter, or matter approaching to it, is deposited about the end of the bone, which is often, if not always, reabsorbed: this will, I believe, frequently engage the extremities of the nerves, and so occasion much inconvenience in that as well as in other ways: indeed, I cannot help thinking that the consequences accruing to the nerves may have been, possibly, too exclusively attributed to diseased thickening, originating in the nerves themselves; for in these cases we observe that, from the position of the wound, the nerves being turned in upon the divided surface of the bone, together with the other soft parts, while that bone pours forth its peculiar effusion in common with them: they thus become entangled in a matter dense and unyielding; into which, moreover, they are pressed by the straps and bandages applied, and hence peculiar causes of irritation are afforded. In other wounds, whatever their nature may be, it is comparatively rare to find such consequences ensue to the nerves.

"This deposit of osseous matter more particularly attracts the attention of the surgeon, as the soft parts are healing, and it is accompanied with the same kind of increased action which is not uncommon in fracture, whether complicated with a wound or not. Similar measures are beneficial in both cases, namely, leeches, and evaporating lotions or poultices, to the immediate seat of this action, (which is denoted by pain, heat, and *solid* swelling;) and blisters applied more remotely." (P. 229.)

There is a case of *Extra-uterine Fœtation,* with remarks, by Mr. Selwyn. The patient was a young woman, aged twenty-eight, who was attacked with vomiting, purging, and tenderness of the abdomen, and sank in about thirty hours.

"About forty hours after death, I examined the body, assisted by my partner, Mr. Miles Astman Wood. On making an incision into the abdomen, a quantity of blood began to escape, and, with a sponge, we removed between three and four pints of that fluid from the pelvic cavity. There was, also, a round tumour, apparently of coagulated blood, lying in it; but on separating the external layers, a fœtus, about three months old, was seen within, floating in its liquor amnii. On examining the uterus, and its appendages, a large ruptured cavity was found (apparently, for I could not trace its canal) in the right fallopian tube, from which the fœtus had escaped. As a drawing will convey a more correct idea of the condition of the parts, than could be given in words, one is subjoined from the pencil of Mr. Ballard, of Ledbury; which he was kind enough to draw, at my request. Neither the peritoneum, nor its enclosed parts, presented any marks of inflammation. The bowels, on the contrary, were particularly blanched." (P. 233.)

From Mr. Middlemore's able essay on *Pterygium*, we must be content with extracting a passage on the treatment.

"We may sometimes arrest the progress of pterygium by the use of astringents and stimulants; and the cases in which they are most usefully employed for this purpose, are, when the pterygium is small and of recent origin, and is actually increasing in size. The common zinc wash, or the nitrate of silver or sulphate of copper drops, in the proportion of two or three grains to the ounce of water, are the best remedies for this purpose; and the patient may be directed to allow a little of the one or the other of these applications, as may be preferred, to be dropped upon the pterygium, from a common capillary tube, two or three times a day. If, by these means, its increase can be checked, then their use may be suspended, (and this is more particularly necessary when the nitrate of silver drops are employed, on account of their tendency to tinge the conjunctiva) for it not uncommonly happens that, when once the progress of pterygium is arrested, it will not again increase, or will at least remain stationary for a long time; still, if at any future period it should evince a disposition to enlarge, the same practice may be again employed.

"Escharotics were formerly much used, but their employment is now pretty generally discarded from our catalogue of remedial measures for the cure or relief of this disease; and very properly so, for, undoubtedly, they are very bad applications; and to them must be attributed many of those troublesome diseases, into which pterygium may degenerate from injudicious and mischievous treatment.

"Scarifications have been frequently employed, and, I may say, successfully employed, for the cure of pterygium; but it is a tedious and painful mode of cure, compared with the operation of excision, that is, the partial excision of pterygium, for its entire removal is, in many instances impossible, always unnecessary, and sometimes very injurious." (P. 256.)

The next paper consists of Dr. Walker's *Observations on the Diseases of Children*, continued from the last volume. He narrates the following cases, which we must give in an abridged form. A girl, aged three years, was recovering from scarlatina, when her abdomen began to increase in size, so that effusion within its cavity was suspected. She was cured by mercurial remedies, iodine liniment, and ioduret of iron. A boy, of sanguineous temperament, afflicted with epilepsy, was not relieved by cupping, blistering, and purging; but a temporary suspension of the fits took place when his mouth became affected by mercury. A girl, aged six years, was recovering from hooping-cough and measles, when she was attacked with paraplegia. Counter-irritants were applied to the spine, but no benefit was derived from them: the brain became affected, and fatuity supervened. Blisters and the tartar-emetic ointment were resorted to, but in vain. Some advantage was obtained, however, from the mercurial treatment: there was less fatuity of countenance, and, from the use of friction, the limbs regained part of their power.

A boy, named George Roberts, was an in-patient of the Huddersfield Infirmary. He was suffering under paralysis of the upper extremities; and his case, while it shows the efficacy of strychnine, will show likewise the caution with which it must be administered.

"This patient had formerly been relieved for a disease of the cervical vertebræ. At the period of his admission he was unable to move his left arm, and had not the free use of his lower extremities; his head was drawn downwards to the left shoulder, and his articulation was indistinct; the pulse was natural; bowels regular; tongue clean.

"Nov. 25th.—A blister was applied to the nape of the neck, and he took gr. iv. of the pil. hydrargyri every night. A stimulating liniment was rubbed twice a day on the back, with the occasional use of the flesh brush, and electricity.

"He continued the pil. hydrarg. until the 18th of December, at which time the mouth was affected, but without any apparent improvement. I therefore had recourse to the strychnine with less reserve, indeed, inasmuch as I was satisfied that the remedy would have a fair trial, under the judicious superintendence of our house surgeon, Mr. Clayton, from whose notes the following account is taken.

"Dec. 18th.—A blister was ordered to the nape of the neck, and to apply to the blistered surface one eighth of a grain of strychnine.

"The blister was kept discharging, and the strychnine applied twice a day, until the 23d of December, when he complained of pain in the bowels, and some feverish symptoms; and, in consequence, the strychnine was omitted, and some saline aperient ordered, which he continued taking until the 4th of January, 1834, when he was in a state to resume the use of the strychnine, in the following pills. ℞. Strychninæ gr. i.; conf. rosæ q. s. f. pil. no. x. Capt. pil. i. bis in dies. Cont. electricitas et frictio.

"Jan. 12th.—Upon the whole better. Appetite improved; no twitchings. To take a pill, with one eighth of a grain, twice daily.

"21st.—Still no twitching, nor any relief in the symptoms. To take a pill, with one sixth of a grain, twice a day.

"25th.—Has had some little pain in the head, which was removed by a smart purgative. ℞. Strychninæ gr. ij. in pil. ix. Capt. i. ter die.

"26th. Some twitchings in the arms last night, and pain in the head this morning. Bowels open; urine copious; pulse regular.

"27th. Pain of the head gone; twitchings the same.

"The strychnine was gradually increased, without any visible amendment, until he took half a grain twice, and then three times a day; but on the 7th of February, about noon, whilst eating his dinner, he suddenly fell from his seat, his right arm forcibly extended, the teeth clenched, pupils immoveable, face of a livid hue, lower extremities quite stiff and stretched out. In this state he remained about twenty minutes. He was not, however, completely relieved for many hours, the same symptoms recurring in a less degree, and he could part with no water. Some brandy and water, with spir. ammoniæ comp. was given as soon as he could swallow, which, with the constant application of warm fomentations to the abdomen, and the use of liniment, &c. eventually relieved him. His bowels were freely opened, and in the evening he passed some urine.

"Feb. 8th. Complains of great weakness and sickness. To continue the ammonia.

"9th. He now parts with his urine as usual, and his bowels are open, though he still complains of some pain, as well as sickness. Pulse seventy-four; heat natural. ℞. Spiritus ammoniæ comp.; spir. lavand. comp. *aa* f. ʒij.; misturæ camphoræ; infus. cascarill. *aa* f. ℥iv. M. capt. ℥i. ter in dies.

"14th. Better in every respect. Rept. mist. et capt. pil. aloes comp. no. ij. h. s.

"24th. Has been gradually improving since the fit, and has regained the free use of his legs and arms. Says that he has occasional pain in his bowels, for which he was ordered the magnesia mixture, with a few drops of tinct. opii.

"On the 28th he felt himself so well, that he was permitted to leave the house. About two months afterwards he appeared to be even better than when he left the infirmary." (P. 279.)

Mr. Ingleby's *Essay on Puerperal Convulsions* is a good summary of what is known on this subject; but, as we have already in this Number given a review of Velpeau on this disease, we shall pass it over, with the exception of three cases.

"Case iv. In this case, which occurred about the sixth month of a first pregnancy, the convulsions were associated with hemiplegia. The muscles of the right side of the face were, at times, convulsed; the right angle of the mouth fell; and the pupil of the right eye was dilated. The patient had difficulty in uttering certain words; the countenance was dull; the mind melancholy, and materially enfeebled. Labour ensued at the ninth month; on the termination of which the disease began gradually to subside, and in three weeks entirely left her. She perfectly recovered, and afterwards had a large family of children, without a recurrence of the disease.

"Case v. A poor woman, subject to convulsive fits, and the mother of several children, applied to me in the seventh month of her pregnancy, with an enlargement of one of the finger joints. She expressed a wish to be bled, on account of a violent pain in the stomach of several days' continuance: but before my pupil could see her, she was seized with convulsions, and had been bled largely by a neighbouring surgeon. Some hours after this she was bled again, to nearly ℥xl. and other curative means were used; nevertheless, the fits, frequently recurring, ended in stupor. The os uteri was found to be closed. She died in a fit, about thirty-six hours after the seizure.

"*Sectio cadaveris.* Abdomen—the uterus and its contents in a natural state; thorax—slight enlargement of the bicuspid valves of the heart; head—considerable effusion of limpid serum between the pia mater and arachnoid membrane, and an effusion of bloody serum in the ventricles, with adhesion of the membranes to the upper part of the spinal canal. I had not an opportunity of seeing this patient after the attack of convulsions. Ought not the membranes to have been ruptured?

"Case vi. Mrs. F. who resided five miles from Bridgnorth, naturally of a spare habit, had become rather plethoric during her third pregnancy. She complained, on Monday morning, the 15th of July, (being within six weeks of the full term of gestation) of sick headach, and went up stairs to lie on the bed. The servants soon heard a heavy fall, and, on entering the room, found her on the floor, struggling violently, quite insensible, and surrounded by blood, which proceeded from injuries received in falling and struggling; this was at ten o'clock a. m. I did not see her until two p. m.; she had then had three fits, and had vomited three times; was partially conscious, but quite unable either to speak, or grasp my coat when I was tying up her arm; the pulse was rapid and very full. The countenance was flushed, except during the convulsion; and the os uteri sufficiently open to admit a finger. After taking away about ℥xvi. of blood a frightful convulsion ensued, which entirely deprived her of consciousness. I allowed the vein to bleed to about ℥xxx., applied cold to the head, and endeavoured, but in vain, to make her swallow some castor oil (the only medicine at hand). She was exceedingly restless, and lay moaning, partly crying, and frequently put her hand to her head. At four o'clock (the earliest moment the medicine could be obtained) I succeeded in giving her eight grains of calomel and a drop of croton oil, administered a colocynth injection, and had twenty-four leeches applied to the temples. The convulsions followed each other in rapid succession; the breathing after each paroxysm became more stertorous, and the coma more profound. At five o'clock the vagina was greatly relaxed, and the os uteri easily admitted two fingers. As the case was assuming progressively a more alarming aspect, I requested a consultation. Mr. Blount arrived at eight o'clock, and acquiesced in the propriety of delivery: at this time three fingers could have passed the os uteri. Rather more than usual difficulty attended the passage of the hand into the uterus, otherwise delivery could not have been more easily or speedily accomplished. No fit came on during the delivery, but another most violent paroxysm ensued immediately afterwards, succeeded shortly by death. The infant survived.

"*Autopsy.* The body was examined twenty-four hours after death. Serum had collected between the arachnoid and pia mater, and also from ℥ij. to ℥iij. in the ventricles. The brain was very firm and healthy. Query: When was the fluid secreted?" (P. 329.)

Dr. Jeffreys has contributed *Pathological Remarks on some Diseases of the Brain, particularly in reference to the Uncertainty of Diagnosis.*

The first case intended to be in illustration of the old thesis, ἡ διαγνωσις χαλεπη, is that of a little girl, who was cured of hydrocephalus internus by leeches, purging, mercurial inunction, and blisters. This was in 1798. She afterwards became the wife of the present Dr. Babington, and died in 1824. He says,

"Your patient, whom you justly represent as having been most interesting, was separated from me by death, in January 1824. Her health had been much weakened by the hot climate of India, and a very severe confinement with her first child; but she lived to give birth to four other children, and had in some measure rallied, when she became the subject of tubercular phthisis, which was developed in consequence of a casual cold, and ran rather a rapid course. There was no post-mortem examination of her remains. She was for many years liable to occasional pains in her head, and to a nervous sensation, which at times prevented her using her needle, or reading, or even playing from written or printed music. She described the sensation to be as if the point of the needle, or the letters of the book, or notes of the music, were advancing towards her eyes, or even touching them; and she found relief in having her temples, especially at the outer orbits, gently pressed. These pains and nervous feelings were rather a matter of remark than complaint: they were never to such a degree as to call for medical treatment. I have often heard my father mention the case, and can testify, on his authority, that neither Dr. Saunders nor himself had any hopes of a recovery." (P. 349.)

In this case, however, the diagnosis appears to have been perfectly correct. The hydrocephalus was cured, but some slight affection of the brain remained.

The second case is headed *abscess of the brain,* and the third *ulcer of the brain.* In the former, the patient suffered from pyrosis and other dyspeptic symptoms, as well as intense headach: delirium followed, and shortly put an end to her existence. In the latter, the patient had long suffered from various diseases, some of which were of a formidable nature: they were accompanied by severe dyspeptic symptoms. Vertigo and great anxiety afterwards supervened; and, some months before his death, he became affected with stupor, torpor of his left leg and arm, eneuresis, and other symptoms of paralysis. It is obvious that, even in the former case, there was reason to suspect some affection of the brain, which was obviously present in the latter one; but it must be confessed that the refinement of diagnosis has not hitherto gone so far as to point out, with tolerable certainty, the existence of an ulcer or abscess in the brain.

In the fourth and last case, the cerebral symptoms were well marked; but the appearances, in Dr. Jeffreys' opinion, were unsatisfactory. The brain was more firm and vascular than ordinary, and "the plexus choroides was of a brown instead of a florid colour, and quite of a *morbid appearance.*" (P. 364.)

Mr. Brayne furnishes a case of *Extreme Enlargement of the Articular Epiphyses of the larger Joints, from Rickets.*

We then come to an *Annual Report of the Birmingham Infirmary for Diseases of the Eye,* by Mr. Middlemore. He has some valuable observations on opacity of the cornea, in one form of which he recommends the use of sulphate of cadmium.

"If a thin layer of inflammatory (lymphatic) deposition exist between the corneal lamellæ, it will produce a dirty bluish-white appearance, such as is often witnessed in the light form of opacity of the cornea: and it is to this condition of disease that the following plan of treatment is more particularly adapted. Let a camel hair pencil be dipped in a solution of the sulphate of cadmium (one grain to two ounces of distilled water) and applied to the cloudy part of the cornea three or four times a day.

"This application produces very little uneasiness; and I have not observed that it inflames the eye, or produces any of those unpleasant effects which sometimes follow the use of the oxymuriate drops. I do not usually advise the solution to be used until the cornea is entirely removed: and I think it unnecessary to continue its use after indications of its commencing disappearance are unequivocally manifest.

"Of course, when an opacity of the cornea results from loss of its lamellar texture, the preceding method of treatment is not likely to be serviceable, except inasmuch as it may cause the removal of a sort of halo, or slight nebula, which surrounds the dense leucoma.

"The treatment of opacity of the cornea by the insufflation of calomel, &c., which has been stated to have been so successfully adopted by Le Pelletier, Dupuytren, Sanson, and other celebrated French surgeons, has not succeeded in my own practice. In fact, the inflammation excited by the insufflation tends to augment the malady; for it must never be forgotten that, when a small opacity of the cornea is present, every slight attack of ophthalmia is liable to increase its size; even a degree of inflammation, which would be inadequate to give rise to lymphatic deposition in any part of the cornea, if none previously existed.

"A weak solution of the oxymuriate of mercury is a favourite and very common remedy for the cure of opacity of the cornea, and there can be no doubt respecting its efficiency; but it produces a good deal of pain, and, when employed too soon, or is injudiciously continued, it is prone to recall the inflammation, the effects of which it is employed to remove." (P. 376.)

The use of the cadmium seems to be adopted from Dr. Tott, whose recommendation of this remedy we gave on a former occasion. (*Med. Quart. Rev.*, vol. i. p. 428.)

The *Reports of the Out-Cases treated at the Birmingham Infirmary*, by MR. PARSONS and MR. RYLAND, are interesting documents; but we can afford space only for a short extract from the latter.

"Fever was very prevalent in Birmingham during the summer quarter: it chiefly affected the bowels, producing ulceration; at least, in every case in which I had the opportunity of examining the body after death, ulcers were found in the cœcum and ileum.

"One of the cases, in which ulceration of the bowels was found, is curious, from the circumstance of the patient dying of hemorrhage that had its source in the ulcers. John Taylor, æt. thirty-four, shoemaker, was seized with fever on the 4th of November. Under the usual treatment he seemed to be improving rapidly, and began to take nourishment, when, in the night of the 16th, he had two large motions, composed almost entirely of blood. There was no particular fulness about the bowels, but pain was felt on pressure being made in the right iliac region.

"17th. The hemorrhage from the bowels continued; and, early in the morning of the 11th, he was found dead, a great quantity of blood having flowed into the bed, unobserved by the attendants.

"*Sectio cadaveris, twenty-nine hours after death.* The body was remarkably exsanguineous throughout. A great quantity of dark fluid blood was found in the colon, the cœcum, and the lower part of the ileon; the rest of the small intestines were pale and healthy. Numerous ulcers were found in the cœcum, and four large ones in the last six inches of the ileon; the latter were deep and sloughy, and it is probable, that from them the hæmorrhage had taken place, which terminated the patient's existence.

A minute examination shewed no other source for the blood." (P. 397.)

Our readers will remember a case something similar to this, narrated by Mr. F. E. Hicks, and inserted in the Med. Quart. Review, vol. iii. p. 457 et seq.

The *Biographical Sketch* of the late Dr. ROBERT JACKSON, inspector of military hospitals, affords a pleasing picture of what zeal and industry may do, unaccompanied by brilliant talents. Dr. Jackson was the first among the moderns who used the cold affusion to any considerable extent, and the first to introduce it into our military hospitals. Perhaps his best work was the one entitled "A View of the Formation, Discipline, and Economy of Armies; with an Appendix, containing Hints for Medical Arrangement in actual War," of which the second edition, in one volume quarto, appeared in 1824. He also wrote in the periodical Journals of the day: e. g. "A Case of Sphacelation of the Intestine," in the London Medical and Physical Journal, vol. vii.; "On the Virtues of the Spider's Web," *idem,* vol. xxi. and vol. xxii.; "On the Virtues of Eye-bright," *idem,* vol. xxiii. &c.

Dr. Jackson appears to have thought highly of the medicinal virtues of cobweb, and asserted that it was more effectual than bark in ague, or than opium in spasmodic diseases. Dr. Barnes, to whom we are indebted for this biography, says, "that not long ago he administered the cobweb, so much praised by Dr. Jackson, in a case of intermittent fever, without the least apparent benefit. The disease resisted both cobweb and quinine, and yielded readily to arsenic. Though it would be wrong to draw any inference from a single case, and that case an obstinate intermittent, it is not improbable that Jackson much over-rated the virtues and efficacy of the remedy." (P. 416.)

Dr. Jackson died of a paralytic affection, in 1827, when he was in the seventy-seventh year of his age.

We wish we could transfer to our pages the whole of Dr. SYMONDS' *Account of the Examination and Appearances of a Corpse, fourteen Months after Death, and of the Detection of Poisoning by Arsenic;* but, as this must not be, we will content ourselves with a few long extracts.

"The face of the corpse was shrunken, and of a dingy yellow colour; the nose depressed; the orbits sunk; the cheeks collapsed and wrinkled; and the mouth looked as if open, in consequence of the retraction of the lips. The appearance, on the whole was not very unlike what would be presented by a wet bladder strained over a skull. The integuments of the trunk had a dull white aspect. The abdomen was considerably flattened, but the thorax had maintained its usual convexity. An incision was made on each side, through the integuments and cartilages of the chest, and continued through the abdominal parietes, and the flap was turned down over the pubes.

"*Abdomen.* The state of the alimentary tube excited the surprise of the by-standers, by its remarkable degree of preservation; every part being almost as distinct as if the inspection had been made at a very short period after death. The peritoneum was smooth, but rather less glistening than usual, and of a duller white. The intestinal canal appeared to contain neither fluid nor gas, and some of the convolutions were matted together. The omentum was firmer than in a recent body. The liver was shrunk to a fourth or fifth of its volume, and was of a very dark colour. The spleen was black, pretty firm, and, when cut into, stained the fingers with a sooty matter. The pancreas had lost considerably in bulk, but it had gained in consistence. Beneath it was found the splenic vein of usual firmness, and of a deep claret hue in its inner membrane.

"Dr. Riley removed the stomach, with the lower portion of the œsophagus, the duodenum, the small and the large intestines, in separate portions; the rectum was taken out attached to the uterus and ovaries. On cutting the duodenum, a bright yellow substance escaped, but in very small quantity: the divided end being carefully compressed by Mr. Kelson's fingers. The parts thus separated were placed in clean vessels, and committed to the charge of Mr. Herapath.

"*Thorax.* The diaphragm was entire, firm, and, in its muscular part, of a darker tint than ordinary. The pleuræ had undergone no visible change; but the left contained a few ounces of very dark red fluid. The viscera were extremely collapsed, and lay at the bottom of the cavity, from which, together with the smooth and shining appearance of the pleuræ, it was inferred that there had been no adhesions during life. The heart was so shrunk and flattened, that it had been accidentally cut across in the process of removing the œsophagus; its lining membrane had a reddish-brown colour, having evidently been dyed by a fluid of the same tint which was contained in its cavities.

"The head was removed by a section between the third and fourth cervical vertebræ, in case it might be required for identifying the individual by the teeth. The hair was of moderate length, brown and grey. The scalp peeled off very readily, as well as all the soft parts of the face, being converted into a thick saponaceous matter. The same adipocirous condition existed in the external parts of the neck. The soft parts of the fauces, and the pharynx with its neighbouring tissues, were greatly decomposed, being soft, in some parts semi-fluid, and of a dirty ash colour. The vertebræ were held together by their ligaments, pretty firmly, but the temporo-maxillary articulation was very loose." (P. 435.)

Adipocire was found on the trunk and extremities; formed, probably, by the water which had entered the coffin. On leaving the churchyard, our author proceeded to the medical school, where Mr. Herapath was commencing his chemical investigations. The stomach

"had been cut open along the lesser curvature, and lay expanded on a clean deal board. The appearance which it presented was very striking. A thick and bright yellow coating, like paint, lay on the mucous membrane, particularly over the pyloric third: but it extended, more or less, with some small injections of unstained membrane, to within two or three inches of the great cul-de-sac. A portion of this yellow matter was dried, then mixed with a little charcoal and carbonate of soda, and put into a reducing tube, and heated. A metallic silvery-looking crust, characteristic of arsenic, soon made its appearance in the upper part of the tube. Mr. Herapath, by heating this crust, in contact with atmospheric air, converted it into crystals of arsenious acid, which yielded the usual precipitates to ammoniacal nitrate of silver, ammoniacal sulphate of copper, and sulphuretted hydrogen. This series of tests was repeated five times, for the satisfaction of various spectators, who went away fully convinced, not only of the detection of arsenic, but also of the skill of the operator. Mr. H. subsequently treated some mixed yellow-tinged matter, washed from the stomach, amounting to seventeen grains, in the following manner: thirteen grains were boiled in nitro-muriatic acid, which decomposed the animal matter, dissolved the phosphates, and the arsenic, and converted the sulphur into sulphuric acid. Ammonia having been added in sufficient quantity to supersaturate this acid, the mixture was acidulated with acetic acid, and filtered. A stream of sulphuretted hydrogen, passed into it, precipitated four grains of sulphuret of arsenic. Mr. H. stated, in his evidence, that the remaining four grains, out of the seventeen grains of mixed substance, would have yielded another grain of orpiment." (P. 439.)

The appearances in the stomach and intestines, after ablution, were unsatisfactory: it was the detection of the poison itself which showed the true nature of the case.

"The first medico-legal enquiry arising out of the above investigation was, whether any thing had been found to indicate the cause of death: and I need scarcely remark, after what has been already said, that the pathological appearances afforded nothing but negative evidence, and that the discovery of the poisonous substance itself was by far the most important fact, with reference to the judicial question. As soon as the metallic crust exhibited itself on the interior of Mr. Herapath's test-tube, information was sent to the coroner, who issued a warrant for the apprehension of the person upon whom the suspicions rested. The only question in this stage of the inquiry was, how far the presence of this substance might be presumed to have been the cause of dissolution. Was it sufficient, *per se*, in the absence of proof of any other deleterious agent, to account for death? Had it been introduced during the life of the individual? The answer to the latter question could not but be affirmative, as the parietes of the trunk were entire; or, if it were possible to conjecture that any one might have been ingenious and diabolical enough to have injected the arsenic by an elastic tube, passed along the mouth and gullet into the stomach, in order, at so remote a time, to support an imputation of poisoning, we still should find it difficult to explain how the matter could have reached the jejunum. The deleterious properties of the substance being admitted, was the quantity sufficient to occasion death? Had it been necessary to have answered this question, with reference to the quantity of arsenic which Mr. Herapath actually produced, the reply would have been all but affirmative. The substance was orpiment: supposing its base to have been arsenious acid, which was converted into a sulphuret by the action of sulphuretted hydrogen in the body, in accordance with actual observations of this kind by Christison, the discovery of five grains was nearly conclusive; or, supposing that it had been given in the form of orpiment, as sold in the shops, the answer would have been the same, since the latter preparation contains more than nine tenths of arsenious acid. But there was no necessity for limiting the question to so small a quantity, because much was wasted in the tests, and in the liquids employed in the ablution of the parts, while a considerable quantity remained incorporated with the tissues. It was thought probable by many, as well as by myself, that there must have been as much as a drachm altogether in the stomach and intestines. When pressed, in my examination, to state what I believed to have been the smallest quantity, I said that I felt persuaded that there could not have been *less* than half a drachm. The substance and the quantity, then, being adequate to a fatal effect, did it actually produce this result? In the absence of any proof whatever that any other fatal cause was in operation, there could be but one answer to this question. Nor do I think that the case would have been different, even had we discovered the traces of organic disease in some vital organ; since the experience of every practitioner must have taught him that the fatal results of such lesions have no determinate time, while the period of action for a poison is far more definite; and, consequently, where both are discovered, it is reasonable to ascribe death to the latter, though there is a remote possibility that the effect of the former might have accidentally taken place before the poison had time to complete its work. But there is scarcely any, even the most acknowledged mortal agent, which is not liable to the exception of such a possibility. On finding the appearances of intense enteritis, the inference that this disease was the cause of death might be questioned, if nothing was known of the history during life, on the possibility that some sudden external cause, such as a concussion, or a stroke of lightning, might have cut off the patient. Such refinements, however, are insufficient to divert the belief from the more obvious conclusion: the highest degree of probability in such cases must amount practically to absolute certainty." (P. 456.)

The case in question (as our readers have guessed ere this,) is the one which furnished the materials for the late celebrated trial at Bristol: the corpse was that of Mrs. Smith, who had been poisoned by Mary Anne Burdock. Arsenious acid may be converted into the yellow sulphuret by the contents of the stomach; but in this instance it was the yellow sulphuret (or, at least, a mixture of that and arsenious acid,) which was bought and administered.

Dr. Symonds tried several experiments on dogs and rabbits and a rat, with sulphuret of arsenic, procured (if we understand him rightly,) from the very parcel from which Mary Anne Burdock had been supplied. He calls it a poison of very great activity; yet these experiments hardly seem to show it. Fifteen grains were given to each of two rabbits, (one of which survived it,) and forty grains to another; thirty grains to a dog, thirty-five to another, and eight or nine to the rat. All these animals died, it is true; but we should call the doses rather large, especially when compared with the dose (a drachm?) which destroyed Mrs. Smith. We are not quite sure, however, that it was with the sulphuret of arsenic from the same parcel that Dr. Symonds performed these experiments; and moreover it appears, from Mrs. Burdock's confession, that she had administered another dose of arsenic to her victim on the previous day.

The last paper is *A Return of Medical and Surgical Diseases, treated at H. M. Colonial Hospital, Hobart Town, Van Diemen's Land,* for the years 1821 to 1831, by James Scott, Esq., Colonial Surgeon. The number of cases in Mr. Scott's tables amounts to upwards of 30,000. He observes,

"Vaccine virus is introduced with difficulty, and soon becomes ineffective. Of syphilis I have seen but few cases (not more than six of the primary symptoms,) in the island; and even those were imported from Sydney and the Isle of France: they were never known to extend.

"For phthisical patients the climate is too variable, and consequently too stimulating, and, when the disease is once fully formed, the symptoms become severe, and the sufferer rapidly sinks.

"From the peculiarity of the climate, scrofula and glandular diseases are rare. Idiopathic intermittent fever, malignant cynanche, smallpox, measles, scarlet fever, hydrophobia, &c. &c. have not been met with in the colony. Hooping cough was once introduced, and, for a short time, extended as rapidly and extensively as in England, but gradually became milder, and in a few months disappeared.

"Parturition is, in general, rapid and easy, and followed by a favourable restoration to health.

"The diseases, both acute and chronic, are generally mild, and of comparatively short duration, and yield more easily to the usual remedies than in any other country where I have practised, or of which I have read.

"The general duration of life is not yet known, but is expected to be high, on account of the mild temperature and other general qualities of the climate, which seem to have a beneficial tendency to preserve the animal functions in order." (P. xii.)

Though the extreme length of this review will show the sense that we entertain of the merits of the volume, we are unwilling to dismiss it with merely praise by implication. These Transactions are among the signs of a new and happier era in the practice of physic; for they are an irrefragable proof of that diffusion of really useful knowledge which, in spite of the opposition of declared enemies, and the dangerous aid of lukewarm friends, is the most prominent characteristic of the age. Let the Provincial Association continue its generous exertions; and at one time we shall see fresh sparks of knowledge struck out by the friendly collision of genius and industry; at another, the Society will perform the humbler but not less important task of carrying each practical refinement of our art (hitherto the splendid monopoly of peopled cities,) into the remotest nooks of the kingdom.

